# Current and Future Novel Treatments for Glioblastoma Multiforme

**DOI:** 10.1155/2014/432195

**Published:** 2014-12-22

**Authors:** Betty Tyler, Francesco DiMeco, Rachel Grossman, Gustavo Pradilla

**Affiliations:** ^1^Department of Neurosurgery, Johns Hopkins University, Baltimore, MD 21231, USA; ^2^Istituto Nazionale Neurologico “Carlo Besta”, 20133 Milan, Italy; ^3^Department of Neurosurgery, Tel-Aviv Medical Center, 64239 Tel-Aviv, Israel; ^4^Department of Neurosurgery, Emory University School of Medicine, Atlanta, GA, USA

After decades of slight to modest changes in outcomes, survival for patients with glioblastoma has shown consistent and sustained improvement. Whether this improvement is due to enhanced imaging technologies, increased diagnostic accuracy, earlier detection, advanced microsurgical techniques, functional tissue preservation, postoperative critical care, targeted radiation, or novel adjuvant therapies is uncertain. What is known is that the field is moving in a more promising direction. In this issue we have selected novel and exciting contributions that represent our current landscape and illustrate new directions, highlighting their opportunities as well as their limitations. The goal of this special issue is to stimulate new understanding and encourage cross-discipline collaborations that will assist in changing the outlook for this disease.

We have invited investigators to contribute original research articles as well as topical reviews that provide updated perspective and present exciting contributions. We have included articles that explore intraoperative technological advances like “*Fluorescence-guided surgery and biopsy in gliomas with an exoscope system*” and combine intraoperative imaging with tissue characterization and biomarker utilization such as “*Intraoperative cerebral glioma characterization with contrast enhanced ultrasound*.” Original contributions presenting improved immunotherapy strategies such as “*Interleukin-13 receptor alpha 2-targeted glioblastoma immunotherapy*” and alternative gene therapy approaches were also included such as “*Newcastle disease virus interaction in targeted therapy against proliferation and invasion pathways of glioblastoma multiforme*.” The role of cancer stem cells in GBM malignancy and the progress made in its diagnostic and therapeutic implications are nicely reviewed as in “*Stem cell niches in glioblastoma: A neuropathological view*.” The impact of the intracranial tumor microenvironment and its pathophysiological implications are also discussed in “*Progesterone induces the growth and infiltration of human astrocytoma cells implanted in the cerebral cortex of the rat*.”

It is our intention that this issue will promote discussion of new topics of interest and stimulate interdisciplinary collaborative efforts to improve outcomes for patients with glioblastoma. New modalities of treatment, characterization of the tumor's microenvironment, developing intraoperative innovative techniques, and targeting tumor-specific receptors are all disciplines on the cutting edge of glioblastoma research, the results of which will hopefully lead to marked improvements in outcome.

*Betty Tyler* *Francesco DiMeco* *Rachel Grossman* *Gustavo Pradilla*

